# Bone health in children with Angelman syndrome at the ENCORE Expertise Center

**DOI:** 10.1007/s00431-023-05231-6

**Published:** 2023-10-13

**Authors:** Karen G. C. B. Bindels-de Heus, Doesjka A. Hagenaar, Sabine E. Mous, Ilonka Dekker, Daniëlle C. M. van der Kaay, Gerthe F. Kerkhof, Ype Elgersma, Henriette A. Moll, Marie-Claire Y. de Wit

**Affiliations:** 1 Dept. of Pediatrics, Erasmus MC Sophia Children’s Hospital, Dr. Molewaterplein 40, 3015 GD Rotterdam, The Netherlands; 2 ENCORE Expertise Center for Neurodevelopmental Disorders, Erasmus MC, Rotterdam, The Netherlands; 3 Dept. of Child- and Adolescent Psychiatry and Psychology, Erasmus MC Sophia Children’s Hospital, Rotterdam, The Netherlands; 4 Dept. of Pediatric Endocrinology, Erasmus MC Sophia Children’s Hospital, Rotterdam, The Netherlands; 5 Dept. of Clinical Genetics, Erasmus MC, Rotterdam, The Netherlands; 6 Dept. of Neurology and Pediatric Neurology, Erasmus MC, Rotterdam, The Netherlands

**Keywords:** Angelman syndrome, Bone health, Bone Health Index (BHI), Longitudinal, Genotype, Mobility

## Abstract

Angelman syndrome (AS) is a rare genetic disorder due to lack of UBE3A function on chromosome 15q11.2q13 caused by a deletion, uniparental paternal disomy (UPD), imprinting center disorder (ICD), or pathological variant of the *UBE3A* gene. AS is characterized by developmental delay, epilepsy, and lack of speech. Although fractures are observed frequently in our clinical practice, there are few studies on bone health in AS. The aim of this study is to investigate bone health in children with AS. In this prospective cohort study, we describe bone health in 91 children with AS visiting the ENCORE Expertise Center for AS between April 2010 and December 2021. Bone health was assessed with the bone health index (BHI) in standard deviation score (SDS) measured by digital radiogrammetry of the left hand using BoneXpert software. Risk factors analyzed were age, sex, genetic subtype, epilepsy, anti-seizure medication use, mobility, body mass index (BMI), and onset of puberty. Children with AS had a mean BHI of −1.77 SDS (SD 1.4). A significantly lower BHI was found in children with a deletion (−2.24 SDS) versus non-deletion (−1.02 SDS). Other factors associated with reduced BHI-SDS were inability to walk and late onset of puberty. Children with a history of one or more fractures (22%) had a significantly lower BHI than children without fractures (−2.60 vs −1.56 SDS). Longitudinal analysis showed a significant decrease in BHI-SDS with age in all genetic subtypes.

*  Conclusions*: Children with AS have a reduced bone health. Risk factors are deletion genotype, no independent walking, and late onset of puberty. Bone health decreased significantly with age.

**Table:** 

**What is Known:** *• Children with neurological disorders often have a low bone health and higher risk of fractures.* *• Little is known about bone health in children with Angelman syndrome (AS).*	
**What is New:** *• Children with AS showed a reduced bone health and this was significantly associated with having a deletion, not being able to walk independently, and late onset of puberty.* *• Longitudinal analysis showed a significant decrease in bone health as children got older.*

**Supplementary Information:**

The online version contains supplementary material available at 10.1007/s00431-023-05231-6.

## Introduction

Angelman syndrome (AS) is a rare genetic disorder characterized by a severe developmental delay, epilepsy, and movement disorders [1, 2]. The estimated prevalence of AS is 1 in 24,000 [3]. AS is caused by the loss of function of the maternally inherited ubiquitin protein ligase E3A (*UBE3A*) gene [4]. AS is an imprinting disorder; the paternal gene for *UBE3A* is silenced in neurons. The loss of the maternal gene can occur due to a microdeletion of the 15q11.2-q13 region, a pathological variant of the *UBE3A* gene, uniparental paternal disomy (UPD), or an imprinting center defect (ICD) [5]. Children with a deletion are known with a more severe phenotype [1, 6, 7].

In 2010, the multidisciplinary ENCORE Expertise Center for AS was established at the Erasmus MC Sophia Children’s Hospital in Rotterdam, The Netherlands [1], and recognized by the National Federation of Universities and the European Reference Network ITHACA. We noticed the occurrence of (multiple) fractures — after minor trauma — in some children with AS. Previous studies showed that children with neurological disabilities might have reduced bone health, so-called secondary osteoporosis [8]. Known associated factors for reduced bone health are genetic predisposition, malnourishment, immobilization, late onset of puberty, use of anti-seizure medication (ASM) and/or corticosteroids, low vitamin D level, and endocrine disorders [8, 9]. Low bone health and fractures in people with AS have not been frequently reported. A case report described a girl with recurrent fractures [10]. Coppola et al. described a cohort of 18 AS patients, all walking, in whom 44% had a low bone mineral density (BMD) on dual-energy X-ray absorptiometry (DEXA). BMD was significantly lower in the group using ASM for a longer period of time and in patients with older age [11]. There was no association with sex, BMI, onset of puberty, or vitamin D level. The study did not report on the occurrence of fractures. An abnormal BMD was also found in 38% of 18 children with idiopathic epilepsy without AS, but decrease in BMD was less pronounced. A possible primary factor of the syndrome itself was suggested [10]. A study in a larger population including a longitudinal perspective on bone health in children with AS was never performed.

The aim of this study was to gain more insight in bone health, its longitudinal pattern, and risk factors for reduced bone health in children with AS that may be amenable to preventative measures to reduce fracture risk.

## Materials and methods

### Study design

This observational study presents prospectively collected data on bone health and associated factors in children with genetically proven Angelman syndrome. All children visited the ENCORE Expertise Center for AS between April 2010 and December 2021. The cohort consists of 150 children of 0–18 years of age, who visited the center at least once. For this study, children with AS based on mosaicism were excluded. Written informed consent was given by the parents of the children. Approval was obtained by the Medical Ethical Commission of the Erasmus MC (MEC-2015-203).

### Data collection

Standardized data were collected during annual visits to the Expertise Center. Bone health was assessed by means of bone health index (BHI) at the age of 4, 7, 11, 15, and 18 years, measured by digital radiogrammetry (DXR) of the left hand. The BoneXpert software program (version 3.0.3, Visiana, Holte, Denmark) was used to calculate bone age. Additionally, BHI was calculated and adjusted for bone age and sex. This describes children’s bone quality as a function of the cortical thickness from 3 metacarpals of the left hand [12]. BHI measures the volume of bone tissue, not its mineral content. BHI will therefore be insensitive to disorders that affect the mineral content, for example, in osteomalacia. But it is sensitive to osteopenia, which is a decrease in the amount of bone tissue [13]. The BHI is described in standard deviation scores (SDS), a BHI-SDS between −2 and 2 is within the normal range (based on Dutch reference data from a healthy population of 531 children between 3.8 and 20.1 years) [12, 13].

To study factors associated with reduced bone health, we examined genotype, sex, onset of puberty, epilepsy, use of ASM, mobility, vitamin D suppletion, and occurrence of fractures. Height and weight to calculate body mass index (BMI) were collected and displayed in SDS, based on Dutch references [14].

Data on puberty stage was categorized into three groups according to Tanner stages (breast and genital development) and age of menarche: (1) early puberty defined as B2 before the age of 8 or menarche before the age of 10 in girls and G2 or testicular volume of ≥ 4 ml before the age of 9 in boys, (2) normal puberty defined as B2 between 9 and 13 years old and age of menarche between 10 and 14 in girls and G2 or testicular volume of ≥ 4 ml between 9 and 13 years old in boys, and (3) late puberty defined as B1 in girls at the age of 13 years or older, age of menarche at the age of 15 or older, and G1 or testicular volume of ≤ 4 ml in boys at the age of 14 years or older [15, 16]. Girls under the age of 8 and boys under the age of 9 without signs of puberty were classified as prepubertal and not included in the association analysis of bone health with onset of puberty.

### Statistical analysis

#### Cross-sectional

All cross-sectional statistical analyses were performed in IBM SPSS Statistics Data Editor version 25 [17]. Children with a UPD, ICD, and *UBE3A* pathological variant were merged into a “non-deletion” group and compared with children in the “deletion” group. The differences between groups were first calculated with unpaired *T*-tests for continuous, normally distributed data and chi-square tests for categorical data. Not normally distributed numerical data were analyzed with a Mann-Whitney *U* test. To test for a possible association between BHI-SDS with BMI-SDS and age at menarche, Pearson correlation was used. Multiple linear regression was used to test for a possible association between BHI-SDS with genotype, mobility, ASM use, BMI-SDS, and onset of puberty, while corrected for age and sex. For cross-sectional analyses, data of the patient’s most recent visit with BoneXpert assessment was used. For variables with missing data, complete case analysis was performed. *P* < .05 was considered statistically significant.

#### Longitudinal

Longitudinal analyses of BHI-SDS data were conducted in R version 4.0.5 [18] using a mixed effects model [19] with age as predictor and genetic abnormality (deletion/UPD/ICD/*UBE3A* pathological variant), sex (male/female), epilepsy (yes/no), and independent walking (yes/no) as covariates. The goodness of fit of a model with random intercepts, random slopes, and a non-linear (natural cubic splines) effect for age was tested. This model allows for almost any form of change in BHI-SDS over time, while accounting for patient-specific baseline levels of BHI-SDS and being robust for missing data and unbalanced time points. In addition, the assumption of equal group sizes is less important in a mixed effects model [20], which gives the possibility to test differences between all four genotypical groups. *P*-values were obtained by *T*-tests for the effect of specific predictors on the outcome and by likelihood ratio tests of the full model with the effect in question against the model without the effect in question. In addition to *P*-values, the Bayesian information criterion (BIC) and Akaike information criterion (AIC) were used as an informant of the model quality.

## Results

Bone health was assessed at least once in 91 of 120 eligible children with AS of 4 years of age or older; 40 children had two or more BHI measurements. Reasons for parents for not having an X-ray were time, behavioral issues of the child, or were unknown. In 5 children, it was not possible to calculate the BHI-SDS at most recent visit due to technical failure of the software program.

### Bone health: cross-sectional

Table 1 shows the characteristics at most recent bone health assessment. The mean age of the children with a deletion was significantly lower than the mean age of the non-deletion children. The children with a deletion had significantly more epilepsy, ASM use, a lower mobility level, and an older age at first independent walking.

**Table 1:** Patient characteristics at most recent BHI visit

**Genetic subtype**	**Deletion**	**Non-deletion**	**Total**	***N***
*N* (% total)	56 (62)	35 (38)		91
*UPD*		*13 (14)*		
*ICD*		*3 (3)*		
*UBE3A*		*19 (21)*		
Sex (m/f)	30/26	19/16	49/42	91
Age at last visit in years (SD)*	10.3 (4.6)	12.6 (4.3)	11.2 (1.6)	91
Epilepsy* (% subgroup)	51 (91)	24 (69)	75 (82)	91
ASM^a^ use* (% subgroup) Valproic acid*	51 (91) *31 (55)*	23 (66) *10 (29)*	74 (81) 41 (45)	91 91
Mobility (% subgroup)				
Wheel chair**	12 (23)	0	12 (13)	
Walking with support**	16 (30)	4 (11)	20 (23)	
Walking independently**	25 (47)	31 (89)	56 (64)	88
Mean age of walking independently in months (SD)*	52.2 (29.4)	41.2 (13.9)	46.3 (22.9)	56
BMI-SDS (SD)	0.67 (1.6)	1.24 (1.3)	0.88 (1.5)	87
Onset of puberty (% subgroup)				
* Prepubertal*	24 (44)	7 (20)	31 (34)	
* Early*	2 (4)	-	2 (2)	
* Normal*	26 (47)	22 (63)	48 (53)	
* Late*	3 (5)	6 (17)	9 (10)	90
Menarche (% subgroup)	9 (16)	7 (20)	16	91
Age at menarche in years (SD)	12.3 (2.6)	11.9 (1.2)	12.1 (2.1)	16
Bone age SDS (SD)	−0.35 (1.4)	−0.23 (1.4)	−0.31 (1.4)	73
Bone age in years (SD)*	9.82 (4.4)	12.28 (3.7)	10.76 (4.3)	86
Vitamin D supplementation	21 (38)	13 (37)	34 (39)	87
Fracture(s) (% subgroup)	14 (27)	4 (13)	18 (22)	82
Age at first fracture in years (SD)	9.7 (5.5)	8.0 (1.7)	9.4 (5.0)	80
BHI-SDS (SD)**	−2.22 (1.3)	−1.02 (1.3)	−1.77 (1.4)	86

*ASM* anti-seizure medication, *VPA* valproic acid, *BMI* body mass index, *BHI* bone health index

^*^Significant difference between deletion and non-deletion (*P* < .05); ^**^Significant difference between deletion and non-deletion (*P* < .001)

^a^Clobazam, clonazepam, ethosuximide, gabapentin, lamotrigine, levetiracetam, oxcarbazepine, ralfinamide, topiramate, valproic acid, and zonisamide

**Fig. 1:**
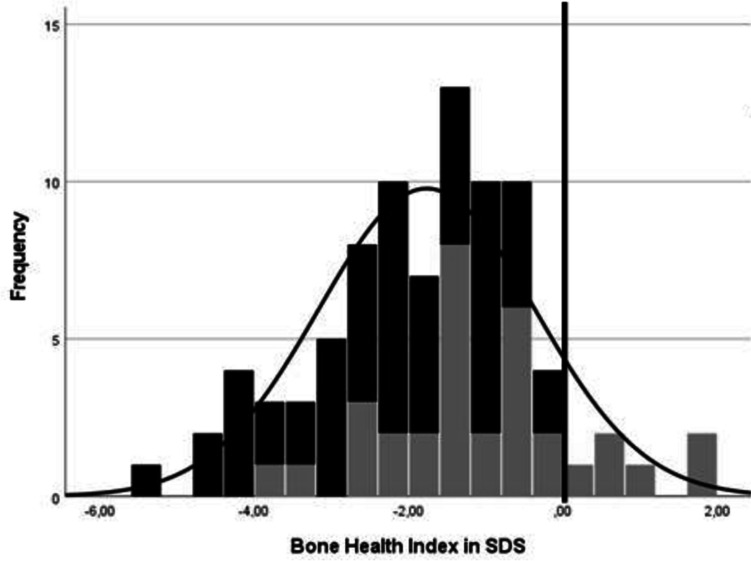
Bone health index SDS in children with deletion and non-deletion. The dark bars display the children with a deletion; the gray bars display children with a non-deletion. The black vertical line represents 0 SDS

Children with AS had a low mean BHI (−1.77 SDS). The mean BHI in the deletion group (−2.22 SDS) was significantly lower than in the non-deletion group (−1.02 SDS). Figure 1 shows a histogram of the distribution of BHI-SDS in children with and without a deletion. There was no difference of BHI-SDS within the non-deletion group.

**Fig. 2:**
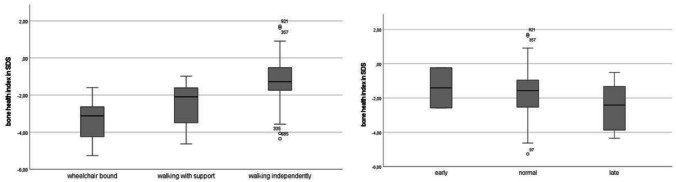
Possible factors associated with bone health: mobility and onset of puberty

Possible factors associated with bone health are displayed in Table 2 and Fig. 2a and b. In addition to deletion genotype, immobility and late onset of puberty were significantly associated with a lower BHI-SDS. Walking independently was significantly positively associated with a higher BHI-SDS. Also, children walking with support had a significant higher BHI-SDS compared to children who were wheelchair bound. There was an association of lower BHI-SDS with ASM use and also with number of ASM use, but this effect disappeared corrected for the other risk factors and covariates.

**Table 2:** Factors analyzed for association with BHI-SDS at most recent BHI visit

		**Mean BHI-SDS (SD)**	**Uncorrected**	**Corrected**^**a**^	***N***
Genotype	*Deletion*	−2.22 (0.2)	**<.001**	**.027**	53
	*Non-deletion*	−1.02 (0.2)			33
	* UPD/IDC*	*−0.93 (1.5)*			*16*
	* UBE3A*	*−1.1 (1.1)*			*17*
Sex	*Male*	−1.85 (1.1)	.579	.439	47
	*Female*	−1.66 (1.7)			39
Epilepsy	*No*	−1.13 (1.5)	**.031**	.380	14
	*Yes*	−1.89 (1.4)			72
ASM	*No*	−1.04 (1.5)	**.026**	.629	15
	*Yes*	−1.92 (1.4)			71
VPA	*No*	−1,24 (1.2)	**<.001**	.476	50
	*Yes*	−2.41 (1.4)			41
ASM number	*1*	−1.57 (1.5)	**.029**	.767	30
	*2*	−1.99 (1.2)			26
	*3 or more*	−2.46 (1.2)			15
Mobility:			**<.001**	**<.001**	
*Walking independently*		−1.23 (1.2)			54
*Walking with support*		−2.44 (1.1)			20
*Wheel chair*		−3.30 (1.1)			10
BMI-SDS			.521	.398	87
Vitamin D	*Yes*	−1.98 (1.7)	.398	-	30
	*No*	−1.70 (1.3)			52
Walking ability duration			.152	-	54
Onset of puberty	*Early*	−1.41 (1.7)	.329	**.036**	2
	*Normal*	−1.78 (1.4)			47
	*Late*	−2.53 (1.4)			9
Age menarche			.404	-	16

*ASM* anti-seizure medication, *VPA* valproic acid, *BMI* body mass index

^a^Multiple linear regression with genotype, mobility, ASM use, BMI-SDS, onset of puberty, sex, and age

Of 82 children, 9 boys and 9 girls (total of 22%) presented with a fracture. Of those 18 children, 13 had a fracture once and 5 had a fracture twice or more. Location of the fractures was wrist/arm in 7, foot in 2, leg in 8, and pelvic in 1 child; no child had clinical signs of a vertebral fracture. Fractures occurred by falling from stairs or jumping at a trampoline in 4 of the 18 children. In 11 children, the fracture was not related to a recalled trauma or related to only minor trauma. In 4 children, the fracture was diagnosed only after a few days of unexplained refusing to stand or crying (see also Table 3 in supplemental file). The children with fractures had a significantly lower BHI compared to children without fractures (*p* = 0.018; adjusted for age).

### Bone health: longitudinal

**Fig. 3:**
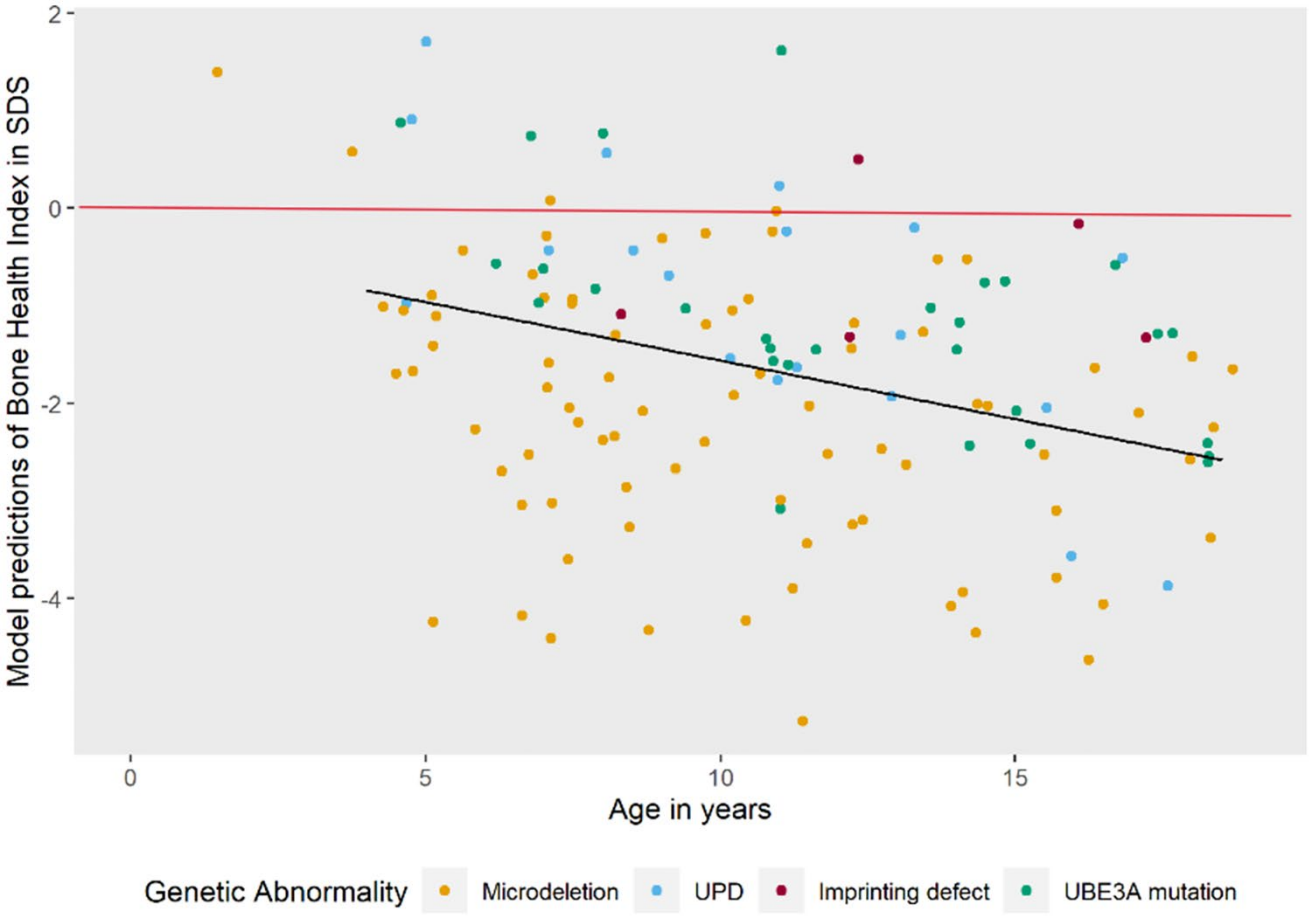
The longitudinal trajectory of bone health index in SDS in AS patients. The black line represents the model predictions of BHI in AS over time, while the red line represents the mean in typically developing children (SDS = 0). The dots represent the individual data points

Data of 134 BHI assessments of 86 children was analysed. The model with the highest fit to the data was a linear model with random intercepts. Figure 3 shows that there was a significant main effect of age on BHI-SDS (*B* = −.12, *t* = −6.22, *p* < .001). As children with AS became older, BHI-SDS significantly decreased. Confirming the cross-sectional analyses, the longitudinal analysis showed a significant effect of genetic subtype and independent walking. The deletion group had significantly lower BHI-SDS than the UPD group (*B* = 1.04, *t* = 2.92, *p* = .005) and the *UBE3A* mutation group (*B* = 0.92, *t* = 2.80, *p* = .006) (displayed in Fig. 4 in supplemental file). Children who could walk independently had a significantly higher BHI-SDS than children who could not walk independently (*B* = 0.98, *t* = 3.57, *p* < .001) (displayed in Fig. 5 in supplemental file).

There were no significant interaction effects between age and genotype (*LR* = 7.68, *p* = .053), age and sex (*LR* = 0.59, *p* = .444), age and independent walking (*LR* = 0.01, *p* = .943), or age and epilepsy (*LR* = 0.01, *p* = .920). This indicates that the trajectory of BHI-SDS over time did not differ between any of these groups. There were not enough data for longitudinal analysis of the effect of onset of puberty.

## Discussion

In a large cohort of 91 children, including longitudinal follow-up, we show that children with AS have a lower bone health than the population reference [12]. Moreover, BHI-SDS decreased significantly with age. Mean BHI-SDS in children with a deletion was below the normal range and significantly lower than in those with a non-deletion. Children with a deletion were younger than non-deletion children at the most recent assessment, which could make this latter finding even more worrying, as BHI-SDS also showed to decrease with age in this study and normally bone mass increases during puberty [21]. The negative effect of deletion subtype persisted when controlled for age and puberty and was confirmed in the longitudinal analysis.

In addition to the deletion genotype, older age, not walking independently, and late onset of puberty were most strongly associated with lower BHI-SDS. We also showed that AS children that walk with support had a significantly higher BHI-SDS than non-ambulatory children. In general, in children with neurological disorders, lack of physical and weight bearing activity is associated with less bone deposition and bone loss and higher risk of fractures [8, 22]. Appropriate physical activities and exercise for children with AS seem important. Since walking with support has a positive effect on bone health, we advise to stimulate the use of walking frames and other walking aids and to keep using them since we know that some children lose the ability to walk independently in puberty [1]. Vertical pressure stimulates bone remodeling [8], so regular use of a standing frame can positively affect bone health in non-ambulatory children. In the general population, the association of reduced bone health with late onset of puberty is established as a physiological phenomenon [23, 24]. When a child with AS shows late onset of puberty, parents may be reluctant to start puberty induction. The negative effect on bone health can be taken into account in shared decision-making.

In literature, use of ASM (in particular use of enzyme-inducing ASM such as phenytoin, carbamazepine, and also the non-enzyme-inducing valproic acid) is indicated as a strong risk factor for low bone health [8, 23, 25, 26]. ASM can activate the CYP450 pathway leading to a lower 1,25(OH)_2_ vitamin D level (carbamazepine, phenytoin, phenobarbital, valproic acid), inhibits osteoblast and stimulates osteoclast activity (carbamazepine, phenytoin, phenobarbital and valproic acid), and/or creates a weak acidosis with decreased bone mineralization (topiramate, zonisamid). A longer period of ASM use might have a stronger negative effect on bone health [8, 25, 26]. Use of three or more ASM induces a stronger risk of osteoporosis than use of one ASM [25]. We found a similar association of BHI-SDS with ASM use in general, with valproic acid specifically, and with the number of ASM. However, these associations disappeared when corrected for age, genotype, mobility, BMI-SDS, and onset of puberty. As mean age in our cohort was 11 years, we could not analyze the effect of long-term ASM use.

In neurotypical children, the prevalence of fractures increases with age with a peak at 11–12 years in girls and 13–14 years in boys. At the end of adolescence, 25 to 40% of the girls and 30 to 50% of the boys had one fracture [27]. The 22% in our cohort is lower, but relatively high if you bear in mind that the mean age of this cohort is 11 years, substantial less children walk, and walk at an older age, putting them at a lower risk to break a bone. Hypotonia, balance problems, and crouch gait, as known to exist in children with AS [1, 2], will make it easier to fall and hamper the ability to break a fall properly, once they walk. In 11 of the 18 children though, the fracture was not related to a recalled trauma or related to minor trauma, suggesting a more fragile bone health.

Neurotypical children show an increase in BMD with a peak bone mass around 25 years for women and 30 for men [21]. As BHI-SDS is already lower in childhood and even decreases with age, we hypothesize that young adults with AS will have a lower peak bone mass than their neurotypical peers. This hypothesis should be studied in future research, by analyzing bone health and fracture prevalence in our patients after the age of 18 years. There is no publication on fracture prevalence in adults with AS beside a general remark on lower bone health in 20% of 53 adults of the cohort study of Prasad et al. [28]. From studies in adults with other neurogenetic conditions with ID, it is known that low bone health and increased fracture risk is prevalent. In a study with 30.522 individuals between 40 and 64 years with ID, low-trauma fractures were seen three times more often than in a non-ID population, with higher age as one of the risk factors [29]. Berkvens et al. followed 136 patients aged 18 to 79 years with ID and epilepsy and showed that 50% had osteopenia and 26.5% osteoporosis assessed with DEXA. During seven years of follow-up, 59% had at least one fracture, of whom 35% had one or more major osteoporotic fractures [30]. Considering our finding of low BHI-SDS in children with AS, we propose the monitoring bone health in adults with AS, performing bone health assessment at least when presenting with a fracture, and starting treatment when osteoporosis is diagnosed.

DEXA is considered the gold standard for bone health assessment [23]. A recent systematic review on measures of bone quality concluded that DEXA and DXR showed the strongest significant correlation [31]. DXR provides information on cortical thickness and metacarpal length and width, representing volumetric BMD, while DEXA is unable to measure bone depth, representing areal BMD. Although future studies are warranted, DXR can be a promising tool to better predict fracture risk [31]. DEXA does not provide information about bone age. DXR is a rapid and easy and therefore more suitable for children with ID and a low-cost measure of bone health.

It is unclear whether the lower BHI-SDS in children with AS has an AS specific origin. Many factors already known to be associated with low bone health are present in AS, such as immobility and ASM use, suggesting that secondary factors likely play a role. But that does not explain why children with a deletion are more severely affected, even after adjustment for mobility and ASM use. Furthermore, it is remarkable that BHI-SDS is already lower than normal at a young age in all children with ASA role for *UBE3A* gene dysfunction with effect on nuclear hormone receptor function and possible effect on vitamin D and hormone function related to bone turnover has been suggested [10]. A recent mouse model study confirmed that UBE3A protein is involved in the nuclear hormone receptor function and cholesterol synthesis [32]. Another mouse model study reports on *NIPA2* positively regulating osteogenic capacity of osteoblasts [33]. *NIPA2* is one of the other genes besides *UBE3A* deleted in AS of the deletion type [5, 34]. *UBE3A* dysfunction in bone is not likely to be a causative factor, as the *UBE3A* gene is only imprinted in neurons. Also, we did not see a difference in bone health between children with AS due to UPD/ICD and a pathological variant of the *UBE3A* gene, and normal expression levels are expected in bone in UPD/ICD children compared to 50% expression in the pathological variant group. Further research is needed to unravel a possible primary AS-specific mechanism for low bone health.

The results of this study combined with previous findings of osteoporosis in adults with ID and epilepsy, which confirm the need for proper guidance and treatment in children and adults with AS. As we are the first to study bone health in a large cohort of children with AS, our data can be used to protocol assessment of bone health in follow-up. We advise to measure bone health at the age of 7, 13–14, and 17 years. At the age of 7, most children showed whether they can walk and/or have manifested epilepsy (and use of ASM). At the age of 13 (girls) and 14 (boys), pubertal development and bone health can be taken into account in the decision to induce puberty, if appropriate. At the age of 17, puberty has completed and assessment provides information for follow-up in adulthood. Assessment of bone health should be considered at any time when a child develops a fracture, especially after non-significant trauma.

Current advice for people at risk for osteoporosis are sufficient sun exposure, suppletion of vitamin D, and optimal nutritional status [8, 23, 35, 36]. Choice of ASM can be considered in the light of their possible negative effects on bone health. Sufficient exercise; maintenance of walking; if needed with support, use of standing table in non-ambulant children; and treatment of late puberty were already discussed. Lastly, when children with AS show unexplained discomfort, even without clear trauma, always consider a fracture. When osteoporosis is officially diagnosed [37], bisphosphonate treatment may be considered [37, 38].

### Strengths and limitations

An important strength of this study is that, to our knowledge, our cohort of 91 children with AS and bone health assessment is the largest described so far. In addition, more than 10 years of follow-up enabled us to also perform longitudinal analyses. Our findings contribute to optimization of follow-up and treatment and thereby quality of life of children and adults with AS. This study also has some limitations. First, there were missing data. Furthermore, data on fractures were partially collected retrospectively, which might have induced recall bias. Lastly, even as BHI closely correlates with DEXA as showed in systematic review [31], it is not the golden standard to assess bone health. Since DEXA is not feasible in most children with AS, we consider BHI as the best possible option.

## Conclusion

We showed that children with AS display low bone health, significantly decreasing with age. Deletion genotype, immobility, and late onset of puberty were significant risk factors. Future studies should focus on follow-up of bone health and fracture prevalence in adulthood, intervention measurements to stimulate bone mass deposition and prevent loss of bone mass with age, and better understanding of underlying molecular-genetic mechanisms. Monitoring and guidance of bone health should become a regular part of clinical follow-up in AS.

### Supplementary Information

Below is the link to the electronic supplementary material.

Supplementary file1 (DOCX 377 KB)

## Data Availability

Data supporting the study findings are available from the corresponding author on reasonable request.

## Abbreviations

ASM: Anti-seizure medication
AS: Angelman syndrome
BHI: Bone health index
BMD: Bone mineral density
BMI: Body mass index
DEXA: Dual-energy X-ray absorptiometry
DXR: Digital radiogrammetry
ICD: Imprinting center disorder
ID: Intellectual disability
SDS: Standard deviation score
UPD: Uniparental paternal disomy
VPA: Valproic acid
