# Supplementary material for: Bone health in children with Angelman syndrome at the ENCORE Expertise Center
Source: Eur J Pediatr. 2023 Oct 13;183(1):103–11. doi: 10.1007/s00431-023-05231-6 (PMC10857954; doi:10.1007/s00431-023-05231-6)
Supplement: Supplementary file 1 — Supplementary file1 (DOCX 377 KB) [file 431_2023_5231_MOESM1_ESM.docx]

**Supplemental file**

**Table 3**

***Circumstances fractures***

| Child | Genotype | Age 1th fracture in years | Location | Circumstances | Minor or major trauma* | Number of fractures | Age of independent walking in years |
| --- | --- | --- | --- | --- | --- | --- | --- |
| 1 | Deletion | 6 | Arm | Tripped during walking | Minor | 1 | 5.3 |
| 2 | Deletion | 11 | Foot | Fall out of wheelchair | Major | 1 | Not walking |
| 3 | Deletion | 7 | Foot | Possible fall out of low bed, fracture 3 days later diagnosed | Minor | 1 | 7..2 |
| 4 | Deletion | 10 | Arm | At the trampoline | Major | 3 | 1.8 |
| 5 | Deletion | 13 | Leg | No trauma recalled, possibly stuck behind foot strap of wheelchair, fracture 2 weeks later diagnosed | Minor | 1 | Not walking |
| 6 | Deletion | 4 | Leg | No trauma recalled | Minor | 2 | Not walking |
| 7 | Deletion | 3 | Arm | No trauma recalled, started crying in bed, fracture 2 days later diagnosed | Minor | 2 | 3 |
| 8 | Deletion | 5 | Arm | Fall from stairs | Major | 1 | 3.2 |
| 9 | Non-deletion | 5 | Leg | No trauma recalled | Minor | 1 | 2 |
| 10 | Deletion | 11 | Leg | Leg got stuck in carousel | Major | 1 | Not walking |
| 11 | Deletion | 11 | Leg | Leg got stuck between spokes of skelter wheel | Major | 1 | 9 |
| 12 | Non-deletion | 7 | Leg | At the trampoline | Major | 1 | 5 |
| 13 | Non-deletion | 5 | Arm | Fall from stairs | Major | 1 | 4.5 |
| 14 | Deletion | 6 | Leg | No trauma witnessed, started crying at daycare | Minor | 1 | Not walking |
| 15 | Deletion | 14 | Hand & wrist | Tripped during walking | Minor | 2 | 3.5 |
| 16 | Deletion | 16 | Leg | Tripped during walking with support | Minor | 1 | Not walking |
| 17 | Non-deletion | 7 | Pelvis | No trauma recalled | Minor | 1 | 8.8 |
| 18 | Deletion | 11 | Arm | No trauma recalled, fracture diagnosed after return from respite care | Minor | 2 | Not walking |

**Figure 4**

*The longitudinal trajectory of BHI-SDS in AS patients per genotype*


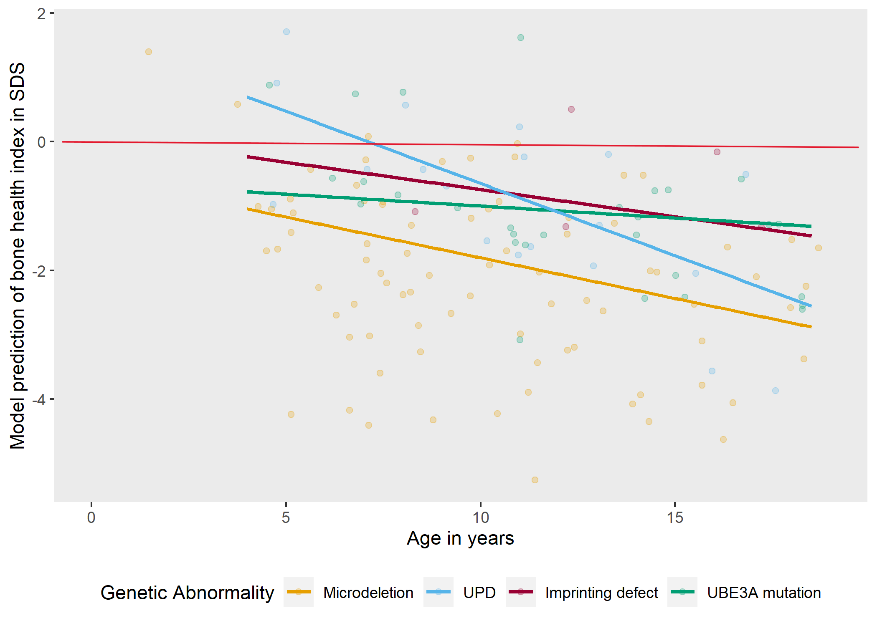


*The yellow line represents the trajectory of BHI-SDS over time for AS patients with a microdeletion, the blue line is the trajectory of BHI-SDS over time for AS patients with an UPD, the purple line represents the trajectory of BHI-SDS over time for AS patients with an ICD, the green line is the trajectory of BHI-SDS over time for AS patients with a UBE3A mutation, and the red line represents the mean BHI-SDS of typically developing children. The dots represent the individual data points.*

**Figure 5**

*The longitudinal trajectory of BHI-SDS in AS patients per walking group*


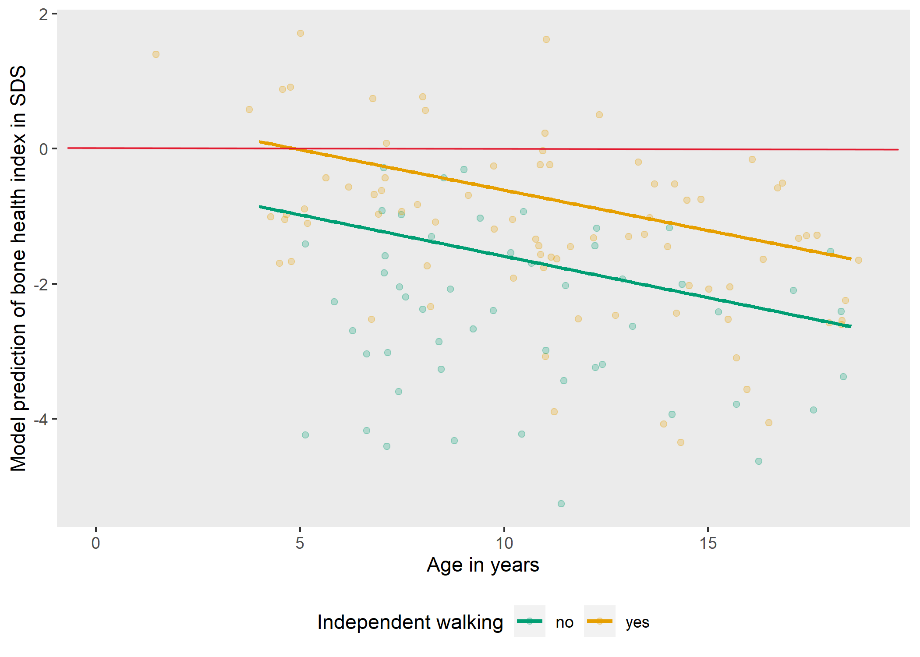


*The yellow line represents the trajectory of BHI-SDS over time for AS patients that walked independently, the green line represents the trajectory of BHI-SDS over time for AS patients that did not walk independently, and the red line represents the mean BHI-SDS of typically developing children.*
